# American Dental Association

**Published:** 1892-04

**Authors:** 


### AMERICAN DENTAL ASSOCIATION.

### DISCUSSION OF DR. KOCH'S PAPER.

(FOR abstract of Dr. Koch's paper, see page 213).

*Dr. Abbott.*—I think the paper is very good, but I do not like the general criticism that is thrown out, that colleges or faculties sell their diplomas for a specified sum of money. I do not think this is fair, and particularly coming from a man who holds no diploma and knows little or nothing about the conduct of colleges. I think he speaks feelingly in his paper. He is in a community of colleges so thick that you can hardly shoot without hitting one or another. But he should not judge all institutions by a few bad ones of his acquaintance.

I believe that the State Boards of Examiners are all very well in their way, but I am convinced that no State Board should be allowed to examine a single man who does not come to them with a diploma from a reputable college to start with. I do not think it is fair to take a man who has been refused by the faculty of a dental college, and then let him practise, after an examination. A law ought to be made to change that in some way.

*Dr. Crawford.*—Outside of the scientific questions pertaining to the art of dentistry, in my judgment the question of legislation is the most important one that addresses itself to the dentists to-day. I shall not speak of the ethical side of the case. I shall look, for a few moments, to the legal aspect of the question. Not a lawyer, but a man who claims to have a limited degree of common sense, I hold the right to my opinion upon a question that I regard of great importance, not only to the dental profession, but to the people of the United States. One plea in the paper was that the people demand it, and that the people were interested in the enforcement of the laws. If you put it upon that plea, I shall certainly claim the question on the other side, because it is a fact that there is not a sufficient number of competent dentists in America to serve the American people ; therefore the discouragement of legitimate institutions of learning is contrary to their best interests, because they are not supplied with a sufficient number of men to fill the want.

I am unfortunate in not having had a college education. I received my education in a local school-house, yet I say that the dental colleges of America should not be abolished. While the people need protection, these institutions need some encouragement. There is a position in this case that I would accept, and that is, let each State in the Union say that any person holding a diploma from a reputable institution of learning shall be admitted to practise without further examination ; but in the same connection giving each board the power to pass upon the diploma that the student holds. That is maintaining the doctrine of State rights which some of us regard so highly.

The law in Tennessee is a model on this point, and I think it would stand a good test,—that when the board has passed favorably upon the college, a man is entitled to respect from a legal standpoint, if he holds a diploma from such an institution. An ulcer upon the body of the educational system will be driven away when this is done in all States, because these boards will pass judgment on those who run disreputable colleges, and say that their practice is not conducted for the best interests of the people.

I think it is the duty of every man to move an injunction against institutions of that kind. Bring it into discussion and you need not legislate it, because it will soon go out of existence on account of the want of patronage.

I hope that the dental profession will deal with it. When the matter was before the Legislature of the State of Tennessee, I asked each member what he thought about it. Every single one of the honorable body of senators said a diploma from a reputable college ought to be recognized. Nine out of ten people whom you ask will tell you so, too; go to any competent juror and see what he says. I hold, Mr. President, that in the views of the people we have the expression of the voice of God. As long as I live in a free country I shall stick to what I think is right, and I shall stand to what I say, though everything on the face of God's green earth should oppose my convictions.

*Dr. Barrett.*—We are all laboring for the same common end, but the great difficulty is that we look upon one side of the question only. The colleges are sometimes viewed with suspicion, because the men are not inside of the workings of the institution, and they suppose it is for the express purpose of making money. It should be urged upon the dental boards that which I believe to be true, that they are working conscientiously for the best interests of the profession, that they desire to stand as safeguards against any out-of-the-way proceedings.

Colleges should provide the instruction, and they should be sustained. What should we do without schools? There is no other method. The boards do not afford education. It seems to me that the trouble is here. Too often there are appointed on the college boards men who have received no curriculum of study whatever. They are unacquainted with the methods of education. They have no knowledge of the principles upon which education is founded. Each school has its own method. They all attain the same end. Every instructor has a peculiar method of his own, by which he reaches the end gained. A board that has not gone through any curriculum of study may desire to ascertain how much an applicant knows. They are not acquainted with the methods of education. They simply ask certain questions. The student comes before them after having passed his examinations and pursued his studies some time before. The board, not having passed through the curriculum that he has, is not acquainted with the methods by which that information has been imparted. They ask strictly technical questions. How many of you could answer them? I cannot do it, because it has passed from my mind. Instead of being able to go through with the methods and educe from the student that which he really knows of the principles of practice, they ask questions which they have committed to memory. That is an injustice. Understand, I do not accuse the boards of doing anything of this kind. It may be the case, however. I believe in the appointment of a national board of examiners. Their duty shall be to examine the State boards and see if they are qualified for their position.

I see I have struck a responsive chord in the heart of almost every one. Say what we will, very frequently these boards themselves have obtained the special legislation through their influence. They have learned the passage of a law which enabled them to sit in judgment upon the schools. That, I believe, is one of the causes of friction. The schools object to being subjected to the criticism and judgment of men who have not passed through the course of study which should qualify other men for such a position. In the first place, no one questions the honesty of the motives. It is said they are looking for money,—that they demand a fee for conferring a diploma. What of it? In the State of New York they demand a fee of thirty dollars for the conferring of every degree. That is an honest charge. They should be compensated for the labor they do; but the point is that they both be made amenable to the same objection. The amount of it is, we should work together. The educational boards and the colleges must come together; they must harmonize their views, or there will be a disruption. There must be an appeal to the courts, and see whether a dental diploma is good for anything or not. A mandamus might be asked for, which should compel a board to show why they should disqualify a student who holds a diploma from a reputable college. They would have a right to such a mandamus and they could obtain it. You see there are these methods of friction. The boards and dental colleges must be brought into harmony. I believe that the office of the college is the more important, because it is the basis of the whole thing in giving the education, which can be obtained at no other hands. Is it anything strange that a college which is organized for such a purpose should object to having judgment passed upon it,—a final, irreversible judgment passed by men who, in many cases, they think are not qualified to do so? That, I believe, is the cause of complaint by the colleges. We know that we have colleges conferring degrees where the qualifications of the student are defective, and if such colleges are to be unquestioned and their diplomas are to be acknowledged upon the part of the community, what would become of our educational law? I believe that the solution might be found to be here: that the boards themselves, who have an important office to fulfil, should examine the methods of teaching. They would thus be enabled to carefully judge of the status of the college, and thereafter recognize the degrees conferred by it.

Now, there is another question. No teaching faculty should confer its diplomas. It is the duty of the teachers to teach, not to grant diplomas. I would have a State board appointed as in the State of New York, before whom should appear the students from all the colleges. Every college should come before it,—medical, dental, literary, or whatever it might be (a different board for each branch of study); before them should appear every student for examination and be found worthy. By this board should be conferred the degrees. Then the schools would devote themselves entirely to teaching, giving their time to the education of youth; all charges of irregularity in the granting of degrees would be removed, and they would no longer be amenable to charges that they are working for money alone.

*Dr. Crouse.*—Many of the societies did not recognize what we wanted when we sent out our circulars. We desired to get at exactly what literary work they had done, and what progress they had made. I speak on that subject, as it was part of the work of this section. It is in connection with the educational part of this subject.

Subject passed.

PUBLISHING THE PROCEEDINGS.

Dr. Abbott moved that the proceedings be published in the journal of the S. S. White Dental Company, and the committee be instructed to endeavor to make arrangements for the publication upon the same terms as previously existed.

*Dr. Truman.*—I think it is time, instead of instructing the executive committee to do as has been stated, that this Association should take into consideration the fact that they ought to publish their own proceedings. I do not believe, Mr. Chairman, that this Association should go to different journals and say, "How much will you give for our proceedings?" It is lowering the standard of our profession. What we ought to do would be to appoint a committee to have them published independently, no matter what it costs; and if they must go to a journal, let them be published in those periodicals that work directly for the profession. The time has come, in my estimation, when this profession should show a professional spirit. It does not show it here, and the fact that this motion was made, that they should be printed in the paper that will pay the highest price for it, is a disgrace to this body. I hope the executive committee will do nothing of the kind; but that they will leave it where it has been before, and I do not see any reason why it should not remain there. It was done exceedingly well; but if there must be a change, let the proceedings be published by this Association.

*Dr. Abbott.*—This is all very well, but the journal that published them the last year did so upon the same terms as the other did, and gave us no better circulation. I agree with Dr. Truman that this Association should publish its own proceedings, and should have them in pamphlet form, one subject after another, as soon as possible. I say if we cannot do that, let us have the journal that will give us the greatest circulation. I know nothing of money matters. I do not care about this question, either. I will help myself to pay for the publication of the proceedings. I want it done at once, as soon as possible, and done well.

*Dr. Cushing.*—I think Dr. Abbott is under a misapprehension in regard to the facts of the case. The proceedings last year were brought out in the smallest time that they ever have been. They were distributed November 4.

*Dr. Abbott.*—I regret that we get nothing of the proceedings until the bound volume comes to us. We lose sight of nearly everything. We have forgotten many of the minor facts; but if they are presented at once, and we have them with us, it becomes of greater interest, and they make more lasting impression upon our minds. I do not care if a dozen journals have the proceedings.

*Dr. Fillebrown.*—In consideration of the exclusive privilege of the reports of this Association, I believe the rule to be that the journal securing the report publishes the transactions. That is the price paid for the privilege. I do not think we get any other consideration.

The motion was then carried, awarding the proceedings to the S. S. White Dental Manufacturing Company.

